# Chemotherapy and quality of life in advanced NSCLC

**DOI:** 10.1054/bjoc.2001.1851

**Published:** 2001-07

**Authors:** D Farrugia, R Lewis

**Affiliations:** Cheltenham General Hospital, Sandford Road, Cheltenham, GL53 7AN, UK; Worcester Royal Infirmary, Castle Street Branch, Worcester, WR1 3AS


*British Journal of Cancer* (2001) **85**(1), 137–140
© 2001 Cancer Research Campaign
doi: 10.1054/ bjoc.2001.1851, available online at http://www.idealibrary.com on IDEAL®

### Chemotherapy and quality of life in advanced NSCLC

**Sir,**
The study reported by Anderson et al (2000) in the August issue of the *BJC* demonstrated a sustained improvement in symptom score with corresponding improvement in QOL, when gemcitabine (GC) was combined with best supportive care (BSC) vs BSC alone in advanced NSCLC. Although GC produced an objective response rate of 19%, with fewer patients requiring radiotherapy (RT); and the time to RT salvage was longer with GC, there was no difference in survival. While this study demonstrates once again that patients' symptoms and QOL do benefit from palliative chemotherapy in advanced NSCLC, it does not eliminate one important factor: the placebo effect associated with receiving intravenous chemotherapy.

It is common experience that patients' expectations from chemotherapy are greater than those of oncology professionals, and therefore patients are more willing to undergo chemotherapy even for small gain (Slevin et al, 1990). The very act of receiving anticancer therapy gives patients a sense of optimism associated with the perception that 'something active is being done' and that they are not just 'wasting away, waiting for the inevitable'. These factors can be very powerful psychological stimuli which lead patients to under-report symptoms either because they truly feel better (placebo effect) or because of underlying fear that reporting toxicity or symptomatic deterioration may lead to early cessation of the treatment.

Where the margin of benefit is so narrow, as in the case of this study of single agent GC in NSCLC, we feel that blinded placebo control trials are required to address the true benefit of cytotoxic chemotherapy in terms of symptomatic improvement and QOL.

De Deyn and D'Hooge (1996), in debating the ethical issues around placebo-controlled trials stated that for such studies to be considered ethical, it was important that no adequate therapy for the disease should exist and/or the presumed active treatment should have side effects. One realizes that the scenario reported by Anderson et al in their study fits De Deyn's criterion rather well.

While we may debate what constitutes an acceptable placebo in such a study, an appropriate 'placebo' may be the same agent given at a sub-therapeutic dose. The placebo arm would then require the same degree of monitoring as the treatment arm in order to confirm that no biological effect is observed on marrow, renal and hepatic function. Only then can we truly evaluate the effect of therapeutic doses of any chemotherapeutic agent on symptoms and QOL, free from observer and patient bias as long as the study remains blinded. And if such a study were to demonstrate equivalence in terms of symptom and QOL benefit, it would have a significant impact on our interpretation of similar studies where chemotherapy with BSC have been compared to BSC alone. Maybe we should use the window of opportunity provided by advanced NSCLC to set up such a study before the opportunity is lost for good.

**D Farrugia**
**Consultant Medical Oncologist,**
Cheltenham General Hospital,
Sandford Road,
Cheltenham, GL53 7AN, UK

**R Lewis**
**Consultant Respiratory Physician,**
Worcester Royal Infirmary,
Castle Street Branch,
Worcester, WR1 3AS

### REFERENCES

Anderson H (2000) Gemcitabine plus best supportive care (BSC) vs BSC in inoperable non-small cell lung cancer – a randomized trial with quality of life as the primary outcome. *Br J Cancer* **83**: 447–453, doi: 10.1054/bjoc.2000.1307
De Deyn P and D'Hooge R (1996) Placebos in clinical practice and research. *J Med Ethics* **22**: 140–146
Slevin M (1990) Attitudes to chemotherapy: comparing views of patients with cancer with those of doctors, nurses and general public. *Br Med J* **300**: 1458–1460

doi: 10.1054/ bjoc.2001.1852, available online at http://www.idealibrary.com on IDEAL®

### Chemotherapy and quality of life in advanced NSCLC – reply

**Sir,**
There are now 5 recent randomized trials assessing the value of chemotherapy versus best supportive care in chemotherapy naive patients with locally advanced or metastatic non-small cell lung cancer. The chemotherapy regimens were single agent gemcitabine, paclitaxel, vinorelbine in a selected elderly population, the combination mitomycin C, ifosfamide and cisplatin (MIC) and single agent docetaxel (Cullen et al, 1999; the Elderly Lung Cancer Vinorelbine Italian Study Group, 1999; Anderson et al, 2000; Ranson et al, 2000; Roszkowski et al, 2000). There was evidence of median or 1 year survival advantage in all studies except the gemcitabine study. However, we note that in the gemcitabine study more patients were WHO performance status 2 (72%) than in the other studies (18–32%), probably because asymptomatic patients were not eligible for entry into our study.

All of the studies have assessed quality of life – a difficult area of research, but all studies have shown some quality of life benefits with chemotherapy. In addition, our study was designed with

